# Expression Profile Analysis of Selenium-Related Genes in Peripheral Blood Mononuclear Cells of Patients with Keshan Disease

**DOI:** 10.1155/2019/4352905

**Published:** 2019-11-17

**Authors:** Xiaojuan Liu, Shulan He, Juanxia Peng, Xiong Guo, Wuhong Tan

**Affiliations:** ^1^Department of Epidemiology and Biostatistics, School of Public Health and Management, Ningxia Medical University, Yinchuan, Ningxia 750004, China; ^2^School of Public Health, Health Science Center of Xi'an Jiaotong University, Xi'an, Shaanxi, China; ^3^Key Laboratory of Trace Elements and Endemic Diseases, National Health Commission, Xi'an, Shaanxi, China

## Abstract

Keshan disease (KD) is an endemic cardiomyopathy, which mainly occurs in China. Selenium deficiency is believed to play an important role in the pathogenesis of KD, but the molecular mechanism of selenium-induced damage remains unclear. To identify the key genes involved in selenium-induced damage, we compared the expression profiles of selenium-related genes between patients with KD and normal controls. Total RNA was isolated, amplified, labeled, and hybridized to Agilent human 4 × 44 K whole genome microarrays. Selenium-related genes were screened using the Comparative Toxicogenomics Database. The microarray data were subjected to single-gene and gene ontology (GO) expression analysis using R Studio and Gene Set Enrichment Analysis (GSEA) software. Quantitative real-time PCR was conducted to validate the microarray results. We identified 16 upregulated and 11 downregulated selenium-related genes in patients. These genes are involved in apoptosis, metabolism, transcription regulation, ion transport, and growth and development. Of the significantly enriched GO categories in KD patients, we identified four apoptosis-related, two metabolism-related, four growth and development-related, and four ion transport-related GOs. Based on our results, we suggest that selenium might contribute to the development of KD through dysfunction of selenium-related genes involved in apoptosis, metabolism, ion transport, and growth and development in the myocardium.

## 1. Introduction

Keshan disease (KD) is an endemic cardiomyopathy with a high mortality rate. It was first reported in Keshan County in northeast China in 1935, while similar cases were found in Nagano Prefecture in Japan and the northern mountains of North Korea in the 1950s. It is characterized by acute heart failure, congestive heart failure, and cardiac arrhythmia. It can be classified into acute, sub-acute, chronic, and latent KD. Currently, acute and sub-acute cases are almost nonexistent, but chronic and latent cases are still scattered in many areas. Most patients are distributed among 2953 townships in 16 provinces from the northeast to the southwest of China, mainly in remote rural areas [1]. Although the incidence of KD has declined in recent years, records still indicate 379,800 chronic KD patients and 1,295,700 latent KD patients, with a total prevalence of 0.13% in China [1].

The etiology of KD has been widely discussed, but it is still unclear. Currently, KD is considered as a gene-environment interaction disease linked to selenium deficiency [2]. Selenium, an essential micronutrient, exerts its function through selenoproteins or small selenium metabolites in the human body [3]. It has been shown that all selenoproteins that have enzymic functions are selenium-dependent, generally with selenocysteine at the active site as a redox center to reduce oxidative damage to biomolecules. In cardiovascular disease, one hypothesis is that selenium deficiency may increase the production of oxidized low-density lipoprotein, thereby promoting the incidence of heart disease [4]. Previous evidence has supported selenium deficiency as a key environmental risk factor for KD. Selenium levels in soil and food are clearly lower in endemic areas than in nonendemic areas, and selenium levels in the blood and hair are significantly lower in KD patients than normal controls [5]. Moreover, it has been reported that the incidence of KD dramatically declined after selenium supplementation [6]. Molecular work has implicated genes related to selenium activity in KD, for example, the selenium-related proteins, glutathione peroxidase 1 (GPX1) and thioredoxin reductase 1 (TrxR1) were found to be decreased significantly in the myocardium of patients with KD compared to controls [7, 8]. However, information is still lacking on expression changes of global selenium-related genes in KD patients. Such data may contribute to understanding of the development of KD.

Blood gene expression profiling has been applied to patients with heart diseases including heart failure and idiopathic dilated cardiomyopathy, and linked to pathological changes as surrogates for disease predication and research [9, 10]. It has been reported that some gene expression signatures in blood can act as valuable diagnostic and prognostic tools for monitoring of disease progression. Moreover, one important pathological change observed in hearts from 308 autopsy cases of KD between 1959 and 1983 is that the tributaries of the intramural coronary system of the left ventricle are closely related to the distribution of necrotic KD foci. Multiple miliary foci usually develop around the branching type of coronary arteries, and the cross section of the terminal arteriole is usually the necrotic focus [11]. In addition to the above, PBMCs appear to hold a great advantage because they are easily obtained for further study or early detection compared to heart tissue. Thus, in this study, we compared the expression profiles of 1517 selenium-related genes in peripheral blood mononuclear cells (PBMCs) of patients with KD and normal controls to identify the key genes that contribute to selenium-induced damage in KD. Both single-gene and gene-set expression analyses were conducted to identify differentially expressed genes and gene ontology (GO) terms, which may provide clues to the pathological mechanism of KD.

## 2. Methods

All studies were approved by the Institutional Review Board (IRB) of Xi'an Jiaotong University. Informed-consent documents were signed by all participants.

### 2.1. Groups and Diagnostic Criteria

Inclusion criteria for patients with KD were the following: (1) KD diagnosis according to “The Diagnostic Standard of the KD in China” (WS/T 210 - 2011); and (2) living in KD areas for more than six months. Chronic KD was diagnosed based on slow onset, cardiac function grade higher than I as per the New York Heart Association, and cardiothoracic ratio higher than 0.55. Latent KD was diagnosed based on insidious onset, cardiac function grade I or normal, and cardiothoracic ratio less than 0.55. Inclusion criteria for normal controls were the following: (1) good health; (2) residence in an endemic area for more than six months; (3) match by age and area of origin with the patients. Patients with other heart diseases and severe illnesses, such as congenital heart disease, diabetes, and hyperlipidemia, were excluded from the study. Based on the inclusion and exclusion criteria, 16 patients and 16 normal controls were selected for the microarray experiment. All were from Xunyi and Huangling counties, Shaanxi Province, China, where the crude prevalence of KD was 1.13% in 2012 [1]. All participants underwent electrocardiography, chest X-ray, and echocardiography. Details of patients and controls are given in Table 1.

### 2.2. Blood Collection and PBMC Isolation

We collected two milliliters peripheral blood from each subject into heparinized vacutainer tubes (Becton Dickenson, San Jose, CA, USA). Blood mixtures were centrifuged at 800 g for 30 min with Lympholyte-H (Cedarlane Laboratories, ON, Canada) to isolate PBMCs, and then rinsed twice using PBS. Cells were stored at −80°C until total RNA isolation.

### 2.3. Extraction of Total RNA from PBMCs

Total RNA was isolated from PBMCs using Trizol reagent (Invitrogen, Carlsbad, CA, USA). Concentration and purity detection was performed by an Agilent 2100 bioanalyzer (Agilent Technologies, Palo Alto, California, USA) and gel electrophoresis containing formaldehyde.

### 2.4. Microarray Hybridization

Isolated total RNA from PBMCs of each patient-control pair was first transcribed into complementary DNA (cDNA), and then reverse transcribed into cRNA and labeled with CyDye using an amino allyl MessageAmp aRNA Kit (Ambion). Before reverse transcription, RNase-free DNase I was used to remove residual genomic DNA. For each patient-control pair, 0.5 μg labeled cRNA was purified separately and mixed together with hybridization buffer before microarray hybridization. Agilent Human 4 × 44 K Whole Genome microarray (G4112F), which consists of 44290 oligonucleotide probes representing 41675 human genes, was employed for microarray hybridization. Hybridization signals were scanned by Gene-Pix 4000B (Axon Instruments Inc., Foster City, CA, USA), Feature Extraction 9.3 software (Agilent Technologies), and Spotfire 8.0 (Spotfire Inc., Cambridge, MA, USA) software were used to record and analyze microarray data. Fluorescent spots that failed to pass the quality control procedures were excluded. Data files were imported into Excel 2010 prior to statistical analysis.

### 2.5. Quantitative RT-PCR Validation

We selected seven differentially expressed genes for validation by quantitative RT-PCR: *ACACB, APOA1, BCL2L1, BNIP3L, CYB5A, PGC-1alpha*, and *HBA2*. *GAPDH* was selected as an internal reference gene. Total RNA samples were prepared in the same way as in the microarray experiment and further converted to cDNA using an RT reagent kit (TAKARA, Takara Biotechnology, CHN). Quantitative RT-PCRs were conducted using SYBR premix Ex Taq™ II (TAKARA, Dalian, CHN) and iQ5™ Real Time PCR Systems (Bio-Rad, CA, USA). We used 2^-△△C(t)^ to calculate relative expression of each individual gene [12]. Paired samples *t*-tests were conducted to compare quantitative RT-PCR data between controls and KD patients using SPSS version 19.0 software, and *P* < 0.05 was considered a statistically significantly difference.

### 2.6. Gene Expression Analysis

To investigate the expression levels of selenium-related genes in PBMCs of patients with KD, we downloaded 1660 selenium-related genes from the Comparative Toxicogenomics Database (CTD, http://ctdbase.org/). After merging these with the normalized microarray data mentioned above, 1517 selenium-related genes (see Supplemental Data [Supplementary-material supplementary-material-1]) were obtained and analyzed by R Studio software. CTD is a publicly available database and provides data as well as detailed steps on “what genes/protein interact with a chemical?”. It provides manually curated chemical-gene interactions in vertebrates and invertebrates from the published literature. Significantly differentially expressed selenium-related genes were defined by a fold change (FC) cut-off of >1.5, a *P*-value < 0.05, and a false discovery rate (FDR) <0.05 [13].

To further identify differently expressed GO terms between patients and controls, we used gene set enrichment analysis (GSEA) software. It provides a normalized enrichment score (NES), which reflects the degrees of over-expression of gene sets, with positive and negative NES values, respectively, indicating the up- and downregulation of gene sets in patients compared to controls. The false discovery rate (FDR) is the estimated probability that a gene set with a specific NES represents a false positive finding. The nominal *P*-value estimates the statistical significance of the enrichment score for a single-gene set. In this study, we choseFDR < 0.25 and *P* < 0.05 to determine significantly differentially expressed GOs. Gene ontology database 6.0 containing 5,917 GO categories was downloaded from the GSEA website (http://www.broadinstitute.org/gsea/index.jsp) for use in this study.

## 3. Results

### 3.1. Quantitative RT-PCR Analysis

**Figure 1:**
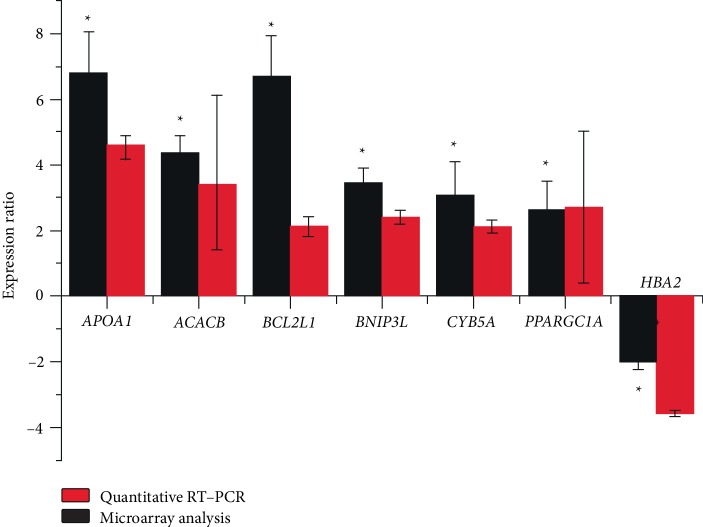
Histogram showing expression levels of seven selected genes as measured by oligonucleotide microarrays and Quantitative RT-PCR.

Quantitative RT-PCR results revealed the increased levels of the following six transcripts: *ACACB, APOA1, BCL2L1, BNIP3L, CYB5A*,* PGC-1α,* and a decreased level of *HBA2*, in KD patients compared to controls (*P* < 0.05). The differences in the relative expression of these genes were consistent with the results of the microarray analysis (Figure 1).

### 3.2. Single-Gene Expression Analysis

We identified 27 differentially expressed selenium-related genes (16 up- and 11 downregulated) in KD patients compared to controls. The 16 upregulated genes were involved in various biological processes, including apoptosis, transcription regulation, metabolism, cytoskeleton and movement, growth and development, and ion transport. The 11 downregulated genes were involved in signal transduction, protein synthesis and modification, cytoskeleton and movement, growth and development, ion channels, and transport proteins (Table 2).

### 3.3. Gene Set Expression Analysis

**Figure 2:**
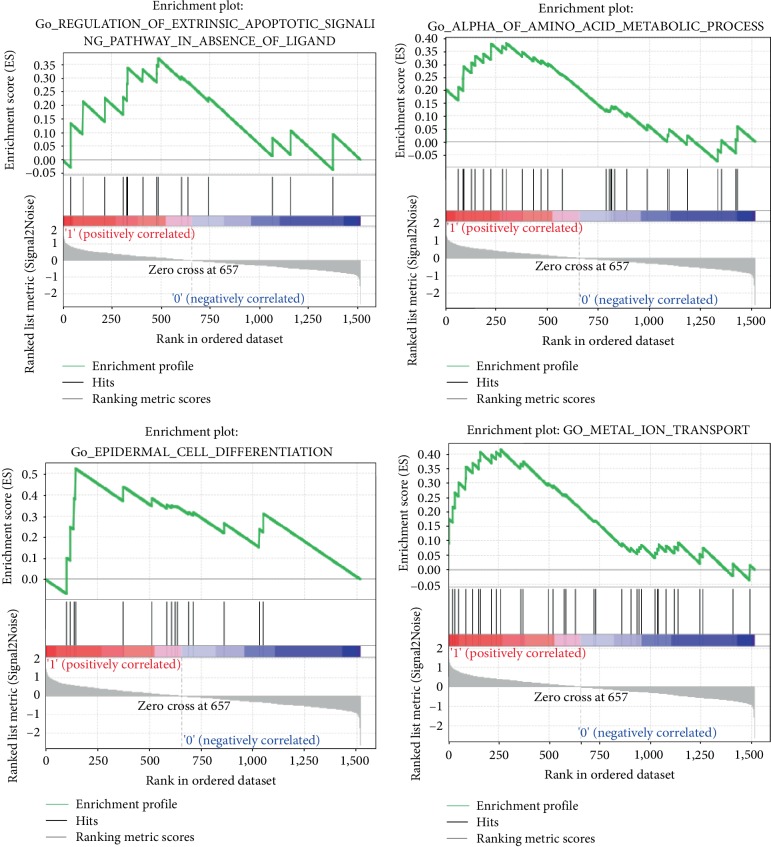
Enrichment plots with the highest normalized enrichment score (NES) for the up-regulated and downregulated genes data.

GSEA detected significant enrichment of multiple GO categories in patients with KD compared to controls. Of these, four were apoptosis-related, two were metabolism-related, four were ion transport-related, and four were development-related (Table 3). The enrichment GO categories with the highest normalized enrichment score (NES) are shown in Figure 2.

## 4. Discussion

In this study, we compared expression of selenium-related genes between patients with KD and normal controls, and identified a set of differentially expressed selenium-related genes and GO categories. Based on the results of this and previous studies, we suggest that selenium deficiency might contribute to KD via effects on the expression and biological function of selenium related-genes involved in apoptosis, metabolism, growth and development, and ion transport.

### 4.1. Apoptosis

Myocardial apoptosis is one of the primary pathological changes seen in KD [14], but the molecular mechanisms underlying this dysfunction, have not been fully elucidated. In this study, we found that the apoptosis-related gene BCL2-like-1 (*BCL2L1*) was significantly upregulated in patients. GO analysis further indicated four enriched apoptosis-related GO categories in patients compared to controls. This result is consistent with a previous study [15, 16], which suggested that apoptosis may play a key role in the mechanism of selenium-related damage in KD.

The outer mitochondrial membrane protein, *BCL2L1* belongs to the anti-apoptotic B cell lymphoma/leukemia 2 (*BCL2*) family. *BCL2L1 *could prevent the release of mitochondrial apoptogenic factors including cytochrome C into the cytoplasm to attenuate apoptosis by regulating the opening of the voltage-dependent anion channels (VDAC). Previous reports have implicated *BCL2L1* and related genes in KD. Evaluation of apoptotic percentage in myocardial tissues from 30 patients with KD (10 acute, 10 sub-acute, and 10 chronic), and five healthy controls, showed that the TUNEL positivity of patients was higher (acute: 2/10; sub-acute: 8/10; chronic: 7/10) than that of the controls [16]. Using Agilent microarray analysis, Xiang et al. identified six apoptosis-related genes that were differentially expressed in KD patients, but *BCL2L1* was downregulated in patients with chronic KD [17]. There is no obvious explanation for this inconsistency, given that the cumulative data indicate the possible roles of apoptosis in the pathological process of KD. In addition to the evidence noted above, selenium deficiency has been associated with upregulated mRNA levels of *BCL-2* and BCL-2-associated X Protein (*Bax*) (*P* < 0.05) [18].

### 4.2. Metabolism

We also found that the metabolism-related genes arginase 1 (*ARG1*), glutamate-cysteine ligase catalytic subunit (*GCLC*), Protease serine 8 (*PRSS8*), and ferrochelatase (*FECH*) were significantly upregulated in patients. GO expression analysis further indicated enrichment of two metabolism-related GO categories in patients compared to controls.

ARG1—which is predominantly expressed in the cytoplasm of the liver, endothelial cells and vascular smooth muscle cells—hydrolyzes L-arginine into L-ornithine and urea. Previous studies have shown that plasma arginase I levels were significantly higher in patients with heart failure than controls [19], and serum arginase activity was found to be significantly higher in 33 KD patients compared with 56 controls [20]. Increased arginase activity in blood of these patients may arise from enhanced spillover of enzyme from damaged myocytes [21]. These were consistent with our results. Increased cardiomyocyte arginase activity in cardiovascular disorders may negatively influence the regulation of contractility, endothelial dysfunction, and heart failure by reduction of NO bioavailability [22, 23]. Additionally, some studies on selenium and arginase activity have been provided. Selenium has no effect on rat liver arginase activity when added to liver in vitro [24]. While another study showed that ARG1 activity was significantly increased when IL-4-treated macrophages were supplemented with selenium and increased activity may contribute to the resolution of inflammation [25]. Thus, it is difficult to explain the mechanisms of dietary intake of selenium on arginase activity in patients with KD using the present data, but it seems that patients with KD supplemented with selenium should not be responsible for overexpression of ARG1 in patients with KD.

*GCLC* is a subunit of the first rate-limiting enzyme of glutathione synthesis. Promoter polymorphisms of *GCLC* have been associated with increased risk of ischemic heart disease in the Kazakhstani population [26]. Uthus and Ross showed that the expression of GCLC was upregulated by selenium deprivation in both rat and mouse liver [27], while increased levels of glutamate-cysteine ligase (*GCL*) may drive glutathione (*GSH*) synthesis in response to oxidants, providing a protective mechanism against oxidative stress [28].

### 4.3. Growth & Development

Another two enriched categories in KD was that of the growth and development-related genes. These included upregulated expression of latent transforming growth factor binding protein 3 (*LTBP3*) and selenium binding protein 1 (*SELENBP1*) and downregulated expression of transforming growth factor beta 1 (*TGFβ1*) in patients with KD compared to normal controls.

There is compelling evidence that *TGFβ1* plays a critical role in various cardiac pathologies involved in heart failure [29]; however, in contrast to decreased *TGFβ1* levels in KD, increased *TGFβ1* levels have been found in the myocardium of human patients with idiopathic hypertrophic cardiomyopathy [30], atherosclerotic, and restenotic lesions [31].* LTBP3* forms a complex with *TGFβ1* protein, and may be involved in the TGFβ1/SMAD3 pathway as a *TGFβ1* target gene [32]. Decreased *TGFβ1* and increased *LTBP3* in patients with KD may suggest a dysregulated TGFβ signaling pathway, which would be a novel component of the mechanism underlying myocardial injury in KD. Further supporting this, Reddi and Bollineni showed that selenium deficiency increases oxidative stress via *TGFβ1* [33]. Thus, selenium deficiency in patients with KD may be an inducing factor for oxidative injury of the myocardium via the TGFβ signaling pathway.

Human *SELENBP1*, a member of the selenium-binding protein family, has been shown to mediate the intracellular transport of selenium [34]. *SELENBP1* is expressed in a variety of tissue types, including heart tissue. Some evidence has shown that decreased *SELENBP1* in lung and colorectal cancer is related to poor prognosis and increased expression level in zebrafish heart, and can be used as a biomarker of aging [35]. Other previous studies may provide clues to understanding the effect of increased *SELENBP1* in KD. Fang et al. found that increasing *SELENBP1* in either human colorectal or breast cancer cells resulted in reduced GPX1 activity, while selenium deficiency was associated with increased *SELENBP1* expression [36].

### 4.4. Ion Transport

The final enriched category we identified in KD was that of ion transport, including ion channel and transport-protein-related genes. Oxysterol binding protein 2(*OSBP2*) and solute carrier family 25 member 37 (*SLC25A37*) were significantly upregulated, while human hemoglobin A2 (*HBA2*), solute carrier family 29 member 1 (*SLC29A1*), ATPase H+ transporting V0 subunit d1 (*ATP6V0D1)*, and solute carrier family 3 member 2 (*SLC3A2)* were significantly decreased in patients with KD compared to normal controls. GO expression analysis results showed that four ion transport-related categories were significantly enriched in patients.

HBA2 is a tetramer of *α* and *β* globin chains. Previous work has shown that the selenium content of red blood cells was significantly lowered in KD, and the activity of methemoglobin reductase was lower in rats fed with grain from KD areas than nonKD areas [6]. SLC25A37 localizes in the mitochondrial inner membrane. The solute carrier family 25 (SLC25) have been shown to transport the molecules include ATP/ADP, amino acids (glutamate, aspartate, lysine, histidine, and arginine), malate, ornithine, and citruline [37]. ATP6V0D1 encodes a component of vacuolar ATPase. Our analysis revealed a significant change in the expression of three members of a solute carrier family including *SLC25A37*, *SLC29A1* and *SLC3A2*, and *ATP6V0D1* may involve in energy transfer in mitochondria [38]. Obvious increases in the numbers of enlarged and swollen mitochondria and significantly decreased levels of oxidative phosphorylation were observed in heart tissue from patients with KD. This might further help to explain the role of energy metabolism disorders in the mechanism of KD.

In conclusion, we identified a set of differently expressed selenium-related genes and GO categories between patients with KD and normal controls. We suggest that selenium might contribute to the pathogenesis of KD through dysfunction of selenium-related genes involved in apoptosis, metabolism, ion transport, and growth and development in the myocardium. These results contribute to increased understanding of the molecular mechanisms underlying KD.

## Figures and Tables

**Table 1:** Characteristics of KD patient-control sample pairs.

		Chip1 (P-C) ^a^	Chip2 (P-C)	Chip3 (P-C)	Chip4 (P-C)
N		4–4	4–4	4–4	4–4
Gender (male/female)		4/0-4/0	0/4–0/4	0/4–0/4	0/4–0/4
Average age (year)		44.75–52.00	41.25–39.74	49.75–48.25	57.75–58.75
N (latent/chronic)		2/2	2/2	2/2	2/2
Heart rate (bpm)		75, 71/54, 62	66, 66/85, 76	82, 75/63, 85	95, 87/62, 64
Cardiothoracic ratio		0.61,0.58/0.46,0.45	0.57,0.58/0.53,0.52	0.63,0.59/0.50,0.50	0.87,0.60/0.47,0.44
NYHA class^b^		Ⅲ,Ⅱ /Ⅰ,Ⅰ	Ⅱ,Ⅲ /Ⅰ,Ⅰ	Ⅱ,Ⅲ /Ⅰ,Ⅰ	Ⅲ,Ⅲ /Ⅰ,Ⅰ
Electrocardiograph		RBBB,ST-T/LBBB, RBBB	ST-T,ST-T/ST-T, ST-T	VPB,RBBB/ST-T, ST-T	AF,AF/RBBB, RBBB
LVEF^c^		0.62,0.74/0.46,0.47	0.48,0.62/0.49,0.72	0.60,0.62/0.77,0.74	0.68,0.65/0.54,0.77

^a^P—C, patient-control.

^b^NYHA class, New York Heart Association class.

^c^Left ventricular ejection fraction.

**Table 2:** Differently expressed selenium related genes in patients compared to controls.

Gene Name	Pubic ID	Function	logFC^a^	*P*-value	FDR	
Up-regulated Genes						
OPTN	NM_001008211	Transcription regulator	1.056	<0.001	0.002	
WASF2	NM_006990	Cytoskeleton and cell movement	1.180	<0.001	0.005	
PRSS8	NM_002773	Metabolism	1.260	<0.001	0.006	
TNFAIP6	NM_007115	Apoptosis	1.199	<0.001	0.006	
SELENBP1	NM_003944	Cell cycle	1.004	<0.001	0.006	
IL1RL2	NM_003854	Interleukin	1.432	<0.001	0.013	
BAG1	NM_004323	Oncogene related	0.898	<0.001	0.019	
OSBP2	NM_030758	Ion channel and transport protein	1.057	<0.001	0.020	
TMC5	NM_024780	Other	0.977	<0.001	0.028	
SLC25A37	AY032628	Ion channel and transport protein	1.121	<0.001	0.030	
FECH	NM_001012515	Metabolism	0.958	<0.001	0.039	
GCLC	NM_001498	Metabolism	0.994	<0.001	0.033	
LTBP3	NM_021070	Growth factor related	0.976	<0.001	0.038	
ARG1	NM_000045	Metabolism	0.938	<0.001	0.046	
CSF1	NM_005211	Cytokine	1.203	<0.001	0.044	
BCL2L1	NM_138578	Apoptosis	1.205	<0.001	0.048	
Down-regulated Genes						
LEP	NM_017526	Receptor	−2.013	<0.001	<0.001	
HBA2	NM_000517	Ion channel and transport protein	-−.698	<0.001	<0.001	
SLC29A1	NM_004955	Ion channel and transport protein	−1.607	<0.001	<0.001	
CSNK2A1	NM_177559	Signal transduction related	−1.668	<0.001	<0.001	
TGFβ1	NM_015927	Growth factor related	−1.278	<0.001	0.006	
RHOBTB3	NM_014899	Signal transduction related	−1.531	<0.001	0.006	
ATP6V0D1	NM_004691	Ion channel and transport protein	−1.127	<0.001	0.019	
SPTAN1	NM_003127	Cytoskeleton and cell movement	−1.118	<0.001	0.021	
SLC3A2	NM_002394	Ion channel and transport protein	−1.092	<0.001	0.030	
CAPNS1	NM_001749	Cytokine	−1.061	<0.001	0.033	
COPE	NM_007263	Protein synthesis and modification	−1.048	<0.001	0.036	

^a^FC, Fold Change.

**Table 3:** Significant enrichment of Gene Ontology categories in KD patients compared to controls.

Gene ontology category	Function	NES^a^	*P*-value
Negative_regulation_of_neuron_apoptotic_process	Apoptosis	1.650	<0.001
Regulation_of_extrinsic_apoptotic_signaling_pathway	Apoptosis	1.665	<0.001
Apoptotic_mitochondrial_changes	Apoptosis	1.534	<0.001
Apoptotic_signaling_pathway	Apoptosis	1.566	<0.001
Cellular_modified_amino_acid_metabolic_process	Metabolism	1.701	<0.001
Alpha_amino_acid_metabolic_process	Metabolism	1.494	<0.001
Epidermal_cell_differentiation	Growth and development	2.084	<0.001
Negative_regulation_of_epithelial_cell_proliferation	Growth and development	1.992	<0.001
Positive_regulation_of_dendrite_development	Growth and development	1.629	<0.001
Gland_morphogenesis	Growth and development	1.615	<0.001
Metal_ion_transport	Ion transport	1.794	<0.001
Cation_transport	Ion transport	1.588	<0.001
Inorganic_cation_transmembrane_transporter_activity	Ion transport	1.527	<0.001
Monovalent_inorganic_cation_transport	Ion transport	1.519	<0.001

^a^Denotes normalized enrichment score (NES) calculated by GSEA. Positive NES indicate gene ontology up-regulated in patients compared to controls.

## Data Availability

All relevant data are contained within the paper. Additional information can be obtained by contacting Dr. Shulan He (hesl@nxmu.edu.cn).

## Supplementary Materials

Supplementary Materials Selenium related genes in gene expression microarray data from Keshan Disease patients and healthy controls.
